# Machine Learning Analysis of Artistic Characteristics for Schizophrenia Classification

**DOI:** 10.1192/j.eurpsy.2024.383

**Published:** 2024-08-27

**Authors:** K. Vasilchenko, A. Leonova, E. Kuznetsova, P. Zdanovich, H. Keren

**Affiliations:** ^1^Human Artificial Control Keren Lab, Bar-Ilan University, Safed, Israel; ^2^Tyumen State Medical University, Tyumen; ^3^Omsk State Medical University, Omsk, Russian Federation

## Abstract

**Introduction:**

Schizophrenia is affecting multiple functions such as cognition, perception, emotion, and social behaviors, and it has also been shown to influence artistic works created by patients. Among the deviations observed in the art works are distinct characteristics like delusional themes, disordered shorter lines, and unique creativity. Such features, along with altered pictorial perceptions and possibly altered motoric function, suggest that it might be possible to differentiate art made by schizophrenic patients from that of healthy individuals. Given the shortcomings of existing diagnostic methods being very long and with a 25% error rate, we proposed a novel neural network model that leverages these artistic markers for classification to support diagnosis.

**Objectives:**

To develop and train a neural network model leveraging unique artistic markers for the classification and support of diagnosing schizophrenia.

**Methods:**

Our study involved 764 participants, 45% diagnosed with schizophrenia, while the others were either healthy or diagnosed with other mental disorders. The average age of the participants was 38.25 years (SD=13.43), and 43.88% of the participants were females. All participants were instructed to draw eight drawings of human faces. These drawings were digitized and categorized based on participants’ schizophrenia status to form the initial training dataset for our model. This data was processed using Python and converted into a NumPy array, which served as input for our model developed using the Keras library. The structure of the model is presented (Image 1).

**Results:**

We used area under curve (AUC), specificity, and sensitivity as key evaluation metrics for our model. The model achieved an AUC of 0.90 on a test dataset that was new to the model and was not used in the preceding training phase. It exhibited a sensitivity of 0.84 and a specificity of 0.85, indicating its capacity to identify schizophrenic and non-schizophrenic individuals, respectively (Image 2).

**Image:**

**Figure:**
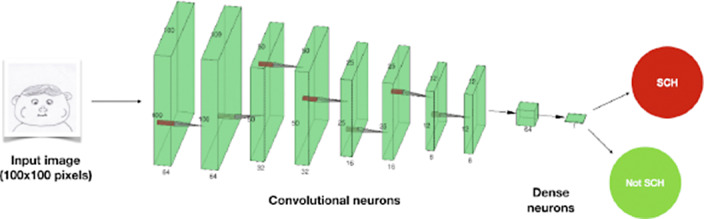

**Image 2:**

**Figure:**
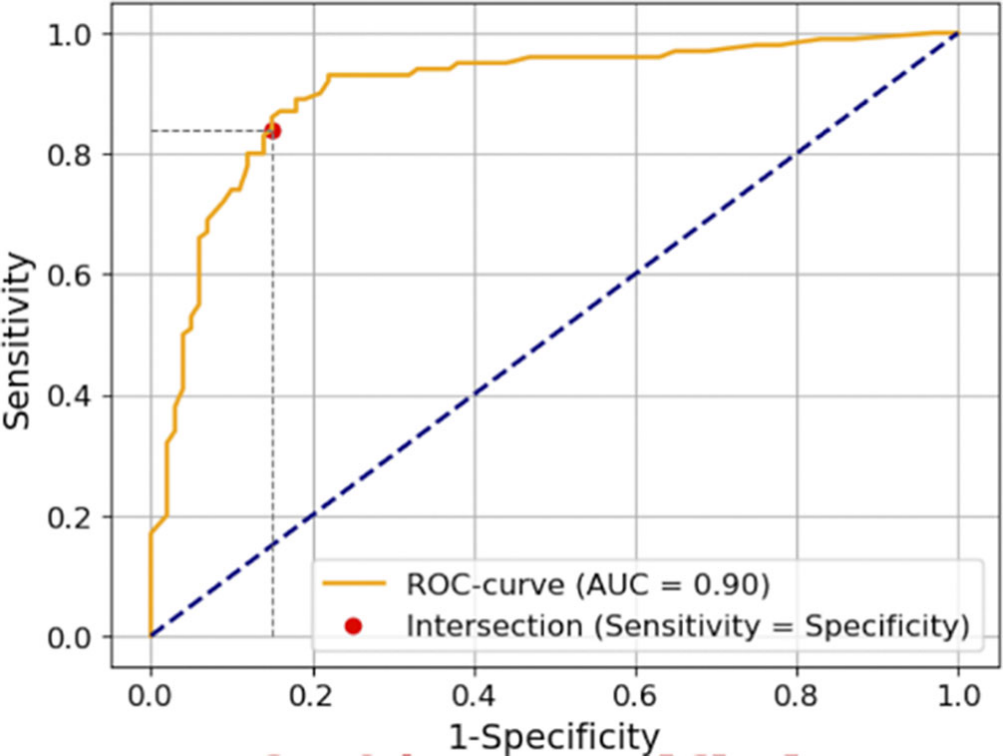

**Conclusions:**

The application of machine learning and AI tools to analyze art created by schizophrenia patients, can offer a promising methodology for exploring the differences between schizophrenic and healthy individuals, as well as a possible support for current diagnostic methods. This approach has the potential to provide an additional fast and more accurate diagnosis, enhancing individualized patient care. Future research will focus on refining and validating the model across diverse populations and various art forms.

**Disclosure of Interest:**

None Declared

